# The Effect of 12 Hour Shifts, Time of Day, and Sleepiness on Emotional Empathy and Burnout in Medical Students

**DOI:** 10.3390/clockssleep1040038

**Published:** 2019-12-06

**Authors:** Lauren A. Fowler, Shannon Ellis

**Affiliations:** University of South Carolina School of Medicine and Greenville, Greenville, SC 29605, USA; SEllis3@greenvillemed.sc.edu

**Keywords:** sleepiness, shift-work, time of day, empathy, burnout

## Abstract

Sleepiness decreases alertness and results in decrements in performance. This is especially problematic in the healthcare field due to restricted sleep from shift-work. Sleepiness increases medical errors, but it also affects emotions and interpersonal interactions. Empathy in physicians is a desirable trait which is associated with increased patient recovery rates and patient satisfaction, and decreased use of pain medication. Shift-work may alter empathy in physicians and affect patient outcomes, but the effects of sleepiness on empathy are unknown. Empathy, which is related to burnout, declines during medical school, while incidence of burnout increases. This study assessed the effect of sleepiness from time of day (TOD) and 12 h shifts on empathy and burnout in medical students. Participants were tested on sleepiness and empathy prior to and immediately following a 12 h Emergency Medical Technician shift. Burnout was assessed following each shift to determine if it was affected by sleepiness, empathy, and shift. TOD affected empathy, with empathy highest in the evening. Sleepiness from working 12 h shifts resulted in decreased empathy and increased burnout, with females showing higher rates on the exhaustion component of burnout. This research demonstrates that TOD affects empathy, and sleepiness decreases empathy and increases burnout in medical students.

## 1. Introduction

Fatigue due to sleepiness is common in modern society, due in large part to working rotating and night shifts in our 24/7 society. Fatigue, due to sleep deprivation, has been shown to be one of the main factors related to many types of accidents, including medical errors [1]. Fatigue and sleepiness are similar, overlapping concepts, but there are distinct differences in how they are measured and defined [2]. Whereas fatigue is defined as a feeling of exhaustion, sleepiness is the tendency or increased propensity to fall asleep, and sleepiness is typically considered to be the inverse of alertness [3,4]. Sleepiness can occur for many reasons, including sleep deprivation, extended wakefulness, and poor sleep quality.

Sleepiness has a detrimental effect on performance, including reducing reaction times, decreasing vigilance, and increasing cognitive and perceptual distortions [5]. The cognitive and psychomotor impact of sleepiness is so great that 17 h of wakefulness leads to cognitive and psychomotor performance similar to someone with a 0.05 percent blood alcohol level (BAC), and after 24 h without sleep, impairment equals that of someone with a 0.10 BAC [6].

While sleepiness-related errors are problematic in a variety of professions, they are especially concerning in healthcare. Numerous studies demonstrate that cognitive performance suffers in healthcare professionals who are sleep deprived, including decreases in memory, vigilance, and spatial abilities [7]. The clinical effects of sleepiness on healthcare professionals is well documented, including Papp’s 2002 study which demonstrated that 84% of the 147 residents surveyed had sleepiness scores that were similar to or worse than those who had been diagnosed with sleep disorders and who required medical intervention for their sleepiness [8]. In addition, Barger (2006), found that interns (first-year residents) reported 300% more fatigue-related adverse events which resulted in a fatality after working more than five extended shifts within a month [9]. These effects of sleep deprivation on performance are not limited only to residents and interns, but the work hours required of those two groups may increase sleep deprivation as compared to other physicians [10].

Medical students have limited access to clinical settings, but sleepiness may affect their performance, too. In an attempt to adjust to their workload and schedule, medical students often reduce their sleep during medical school, and almost 60% of medical students have poor sleep quality [11]. This poor sleep quality can be detrimental to academic performance in medical school [12].

Empathy is the experience of “feeling” what another is feeling, essentially bridging the gap between the experience of self and the experience of others [13]. Empathy is an important attribute for physicians, as it allows them to understand the patients’ experiences and perspectives. In addition, when a physician is empathetic, it leads to increased patient satisfaction and compliance with treatment, improved health consequences, expedited recovery from surgery, and decreased use of pain medication [14]. From the physician side, increased physician empathy results in higher supervisor ratings of clinical competence, decreased malpractice litigation, and increased job satisfaction [15]. However, research has shown that as medical students advance in their medical education and progress toward becoming physicians, there tends to be an overall decrease in empathy [16].

Physician engagement and empathy is considered necessary for mutual physician-patient satisfaction [17,18] and this “clinical empathy” is now characterized as having two major components, one affective and the other cognitive. The cognitive component of clinical empathy is the ability to understand and view the world from the patient’s perspective, while the affective component of clinical empathy is the ability to experience the feelings of the patient [19]. This is similar to Preston and De Waal’s classification of empathy as being comprised of both cognitive and emotional processes [20].

While sleepiness can affect cognition and performance, there are numerous studies to demonstrate that it can also affect our perception and emotional regulation. Sleep loss and poor sleep quality adversely affect mood, leading to increased frustration, a tendency to blame others for problems, and anger rumination with a lower indication for forgiveness [21,22]. Sleep deprivation increases the propensity of the individuals to experience negative emotions [23], and even one night of sleep loss impairs the ability to share the emotional state of others [24] and the ability to process emotional information [25]. While extensive research has explored the effects of sleepiness on cognitive and motor performance, sleep deprivation’s negative effects on mood seem to be more profound than either of those [26].

Sleepiness has also been shown to be associated with the experience of burnout, where empathy may be protective against the experience of burnout [27]. Burnout is a psychological syndrome that is increasing in physicians-in-training and in practicing physicians. In the United States, burnout is approximately two times as prevalent in physicians as compared to workers in other fields, even after controlling for work hours [28]. Physician burnout is positively correlated with depression [29], a 25% increased odds of alcohol abuse/dependence [30] and an increased risk of suicide [31].

Burnout has three main components: a state of emotional exhaustion, depersonalization or cynicism and lower personal accomplishment [32]. Physicians with emotional exhaustion often feel “used up” at the end of the day, and they feel that they cannot share any more of themselves, emotionally, with their patients [33]. Depersonalization is when physicians begin treating patients less like people and more like objects. Lower personal accomplishment involves feeling as if the work you do has no value and is often related to perceived control of the work environment. Repeated-periods of sleep loss, disturbed or fragmented sleep, and shift work can drastically increase the incidence of burnout [34]. A two-year study of more than 300 workers found that insufficient sleep was the main risk factor for burnout development [35].

While sleep loss has been shown to affect some aspects of empathy [36,37,38], Guadagni was the first to recently show that sleep deprivation and poor sleep quality over time can affect emotional empathy [24,39]. However, no known studies investigate the role of acute sleep deprivation due to shift-work on empathy, nor how the time of day of testing affects empathy. In addition, few studies combine the relationship between sleepiness, empathy, and burnout, especially in medical students. This study was designed to assess how 12 h shifts and the resulting sleepiness affect emotional empathy in medical students. In addition, this study examined how sleepiness, time of day, and shift-work are related to levels of burnout in preclinical medical students.

## 2. Results

Thirty-three first-year medical students (15 male and 18 female) participated in this study, but three were excluded due to incomplete data, resulting in 14 male and 16 female final participants. Nineteen of the participants worked day shifts, and 11 worked night shifts. Ages of the students ranged from 22 to 38 years, with a mean age of 24.76 (standard deviation 3.35).

### 2.1. Descriptive Statistics

Descriptive statistics for the variables of sleepiness (Stanford Sleepiness Scale, or SSS), empathy (Toronto Empathy Questionnaire, or TEQ), and burnout (Maslach Burnout Inventory, Student Survey, or MBI-SS) by time of day, shift and gender can be found in Table 1. Pre and post refer to testing periods before and after 12 h shifts.

### 2.2. Inferential Statistics

A 2 (Shift) × 2 (Time of Day) × 2 (Gender) mixed multivariate analysis of variance assessed the effects of shift (day/night), time of day of testing (am/pm) and gender (male/female) on sleepiness, empathy, and burnout. Shift and time of day were tested within-subjects, and gender was a between-subjects analysis. All requirements for Analysis of Variance were met. As expected, sleepiness (as reported by the SSS) was higher after completing the 12 h night shift than after the 12 h day shift, *F*(1,26) = 6.71, *p* < 0.05. The 12 h shift itself did not have an effect on empathy, separate from time of day and sleepiness. There was no significant effect of shift on burnout.

Time of day was grouped as AM (between the hours of 0500 and 0900) and PM (between the hours of 1700 and 2100). These times corresponded to the beginning and ending of the 12 h shifts, which was when data collection occurred. Time of day affected empathy levels, with the PM time showing higher levels of empathy, but only prior to the 12 h shift (*F*(1,29) = 7.497, *p* < 0.01, indicating an interaction between time of day and shift. Time of day did not have a significant effect on burnout.

**Figure 1:**
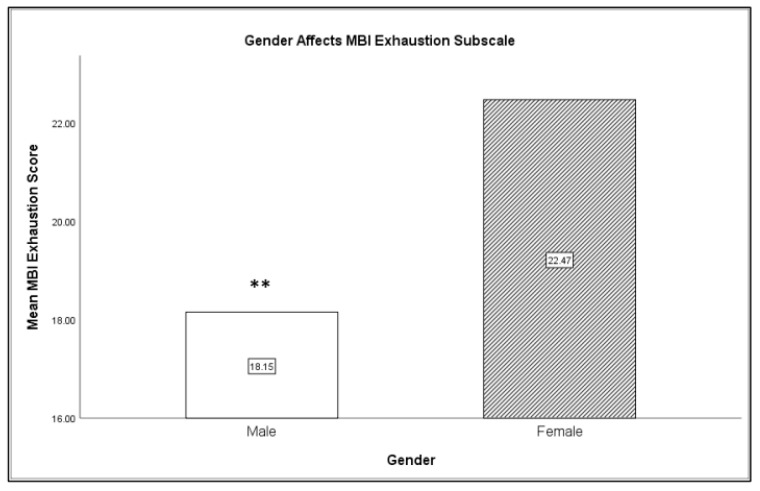
Females reported higher exhaustion as compared to males (** *p* < 0.01).

Gender was classified according to participant choice as either male or female. Gender had a significant effect on burnout, specifically on the personal exhaustion category of burnout. Females had significantly higher levels of personal exhaustion than males, *F*(1,28) = 7.71, *p* < 0.01 (refer to Figure 1). In addition, gender had an effect on sleepiness, with females reporting higher levels of sleepiness overall than males, *F*(1,28) = 3.87, *p* < 0.05. However, males were more fatigued after completing a shift, regardless of time of shift, than females, *F*(1,26) = 4.69, *p* < 0.05. There was no significant difference in empathy levels between males and females.

**Figure 2:**
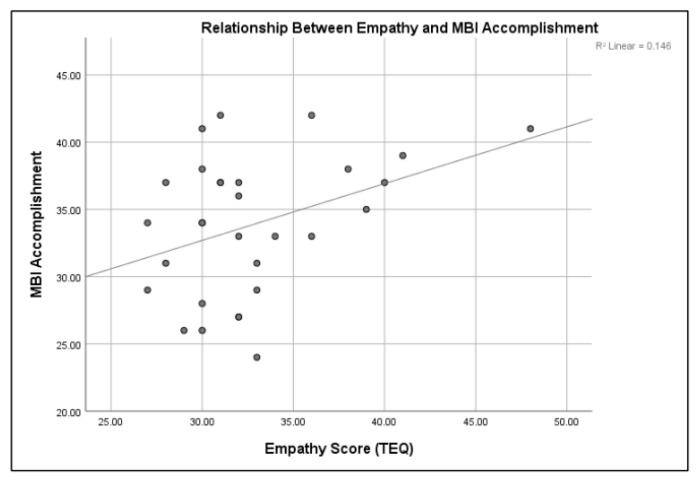
Sleepiness is directly related to personal accomplishment (*p* < 0.05).

**Figure 3:**
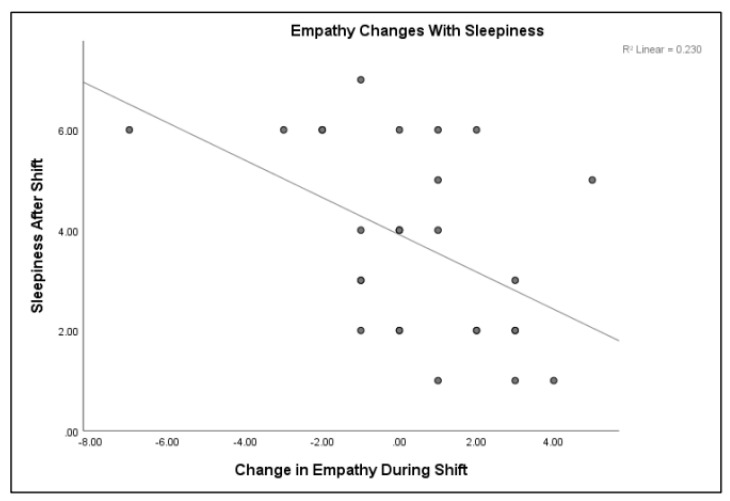
Sleepiness is inversely related to empathy (*p* < 0.01).

A Pearson correlation was conducted to measure the strength of the linear association between burnout, sleepiness, and empathy. Burnout, specifically the depersonalization/cynicism subset, was directly related to post-shift sleepiness, *r*(30) = 0.444, *p* < 0.01. The sleepier the person, the more likely they were to report a feeling of depersonalization, but only after completion of the shift. Burnout and empathy were also related. The personal accomplishment subset of the MBI was directly related to pre-TEQ empathy scores. The higher the sense of personal accomplishment, the higher the empathy score prior to the shift, *r*(30) = 0.387, *p* < 0.05 (refer to Figure 2). This was only true prior to the shift. After the shift, regardless of time, there was no significant relationship between accomplishment and empathy. Empathy and sleepiness were inversely related. The sleepier the medical students (higher score on the SSS), the lower the empathy scores on the TEQ, *r*(30) = −0.480, *p* < 0.01 (refer to Figure 3).

## 3. Discussion

This study assessed the effects of shift (Day, Night), time of day (AM, PM), and gender (Male, Female) on sleepiness, empathy, and burnout. As predicted, working a 12 h night shift resulted in enhanced sleepiness as compared to a 12 h day shift. This is not surprising, given that the medical students typically attend classes and/or study during the day. What was new and notable was that the sleepiness associated with the shift resulted in differences in empathy. The sleepier the participant after working the 12 h shift, the lower their emotional empathy score. This represents the first known work to demonstrate that sleepiness due to shift-work impacts emotional empathy levels.

In addition, empathy scores were different depending upon time of day in both within and between subject analyses, with PM time of day showing higher levels of empathy. However, empathy levels were affected by time of day only prior to working a 12 h shift. Once the shift was worked, empathy levels were no longer significantly affected by time of day. This demonstrates that working a 12 h shift, in addition to sleepiness, results in a decrease in empathy.

While time of day was only assessed at two time points, which is not ideal, it demonstrated two distinctly different levels of empathy in medical students. Future research should focus on time of day and look to see if empathy is at all related to biological rhythms fluctuating with circadian rhythmicity. Kleitman (1938) demonstrated a systematic link between cognitive performance and core body temperature over 75 years ago [40]. It is possible that empathy mirrors other cognitive factors and fluctuates with other physiological factors.

Many studies show that women tend to be better at recognizing facial and body expressions of emotion, as well as showing higher cognitive and affective empathy [41]. While there were some slight gender differences in clinical affective empathy in this study, the difference was not significant. However, this study demonstrated that gender influenced sleepiness. Women reported higher levels of sleepiness than men, which was similar to Akerstedt’s findings that disturbed sleep and sleepiness were more common in women [42]. Although women had higher levels of sleepiness overall, men reported enhanced sleepiness due to 12 h shifts. Gender also had an effect on burnout. Women had significantly higher levels of the emotional exhaustion component of burnout than men.

Empathy and burnout were related, where people who scored higher on personal accomplishment had higher empathy scores, and people who had higher exhaustion levels had lower empathy scores. Both of these subsets of the burnout inventory were affected with shift-work, such that shift-work lowered personal accomplishment and elevated exhaustion levels, both of which lowered empathy levels.

This study was limited by the small sample size (34) and incomplete data from some of the subjects. Subjective sleepiness, as assessed by the Stanford Sleepiness Scale, was used to determine sleepiness/alertness in medical students, but no physiological factors were assessed. Since self-report is notoriously unreliable, future research should include objective measures of sleepiness in addition to self-report.

This study represents the first known research to demonstrate that sleepiness due to shift-work and time of day affects emotional empathy. In addition, this research furthers the work by Guagdani (2014, 2018), Minkel (2010), and Kahn (2013) that demonstrates that sleep loss and sleep deprivation affect emotional interpretation [23,24,25,39]. This is also the first study to demonstrate that sleepiness results in decreased emotional empathy in a clinical setting. Empathy is a valuable skill for healthcare professionals, and it begins to decline as early as the third year in medical school. Based upon this research, more attention should be paid to preventing declines in empathy in healthcare professionals, especially females, as a result of sleepiness and shift-work.

## 4. Materials and Methods

Thirty-four first-year medical students from a southern, urban medical school in the United States were participants. Four participants did not complete all requirements within the time frame required for the study, so their data were excluded. As a part of their medical school curriculum, the students complete Emergency Medical Technician (EMT) training prior to time spent on undergraduate medical education. During their first two years of medical school, the students are expected to participate in monthly 12 h EMT shifts that they work concurrently their classroom and testing requirements. These shifts can be day shifts, starting at anywhere from 0500 to 0800, or night shifts, starting at anywhere between 1700 and 2000 (working 12 h shifts, with 0500 shifts going until 1700, 0600 going to 1800, etc.). The shifts were worked during the medical school year, overlapping with required duties of the students (such as classes, clinical duties, and studies).

Sleepiness was assessed using the Stanford Sleepiness Scale (SSS). The SSS is a self-report Likert scale in which participants are asked to indicate which level of sleepiness/alertness best describes their current state [43]. The options range from (1) “Feeling active, vital, alert, or wide awake” to (7) “Almost in reverie; sleep onset soon; lost struggle to remain awake” [43,44]. A score above “3” is considered “sleepy”. The SSS is one of the most widely used instruments for measuring subjective sleepiness, and it has been shown to be sensitive to sleepiness induced by sleep deprivation [45,46].

The Toronto Empathy Questionnaire (TEQ) is made up of 16 questions that cover a variety of attributes associated with empathy [44]. Items on the TEQ specifically target the perception of emotional state in others and the corresponding emotion in oneself that is elicited, which corresponds to Preston and de Waal’s (2002) emotional empathy and Shamay-Tsoory’s affective component of clinical empathy (2011) [19,20]. The TEQ has been shown to have high test-retest reliability, high content validity [44], and to be highly correlated with the 80-item Empathy Quotient [47]. The TEQ was used to assess emotional empathy in the participants.

Burnout was measured using the Maslach Burnout Inventory Student Scale (MBI-SS) [48]. The MBI is a self-report, Likert scale in which respondents rate the frequency in which they experience various feelings or emotions (ranging from never to daily) [33]. The student scale of the MBI assesses three domains related to burnout, including depersonalization (defined by Schaufeli, 2002 as a cynical attitude of withdrawal and detachment), exhaustion, and personal accomplishment [48]. Higher levels of depersonalization/cynicism and exhaustion and lower levels of personal accomplishment indicate burnout [33,48]. The MBI-SS is the most widely used measure of burnout in students [49].

Institutional Review Board approval was obtained from University of South Carolina (IRB#Pro00084476) to conduct research with the medical students. Participants were asked to volunteer to participate in the study through email recruitment, and all students who consented to participate received a link for participation. All data were collected using RedCap (Research Electronic Data Capture) on an electronic device. All participants were required to complete the tasks within one hour prior to the start of their shift and within one hour of the end of their shift. Any participant who failed to complete the survey within the hour had their data excluded.

Participants were assessed on empathy (using the TEQ) and sleepiness (using the SSS) prior to and following their shift, and they were assessed on burnout following the shift only (using the MBI-SS). Participants were not tested twice on the MBI-SS because, although it has high test-retest reliability [33,50], there are no studies demonstrating that testing within 12 h does not create a testing effect and alter the validity of the instrument.

## Figures and Tables

**Table 1:** Means (standard deviations) of each variable assessed.

	Sleepiness		Empathy		Burnout		
	Pre	Post	Pre	Post	Exhaustion	Cynicism	Accomplishment
**Time of Day**							
**AM (*N* = 30)**	2.27 (1.55)	4.45 (1.81)	31.26 (3.41)	35.27 (6.13)	19.81 (6.30)	9.00 (3.29)	34.91 (5.80)
**PM (*N* = 30)**	3.12 (1.49)	3.29 (1.83)	35.5 (5.23)	34.42 (3.46)	21.58 (5.26)	9.53 (4.44)	33.35 (4.89)
**Time of Shift**							
**Day (*N* = 19)**	3.00 (1.49)	3.21 (1.84)	31.47 (3.45)	32.57 (3.47)	21.16 (5.47)	9.79 (5.03)	33.74 (4.66)
**Night (*N* = 11)**	2.36 (1.63)	4.72 (1.61)	35.18 (5.62)	35.00 (6.26)	19.63 (6.32)	9.00 (3.28)	34.09 (6.11)
**Gender**							
**Male (*N* = 12)**	2.08 (.86)	3.84 (1.99)	32.69 (4.44)	33.85 (3.93)	18.15 (3.69)	9.31 (4.42)	34.85 (4.78)
**Female (*N* = 16)**	3.29 (1.76)	3.71 (1.86)	32.82 (4.92)	33.18 (5.36)	22.47 (6.40)	9.65 (4.55)	33.12 (5.41)

## Abbreviations

SSS	Stanford Sleepiness Scale
TEQ	Toronto Empathy Questionnaire
MBI-SS	Maslach Burnout Inventory-Student Survey
